# Effect of probiotic treatment on the clinical course, intestinal microbiome, and toxigenic *Clostridium perfringens* in dogs with acute hemorrhagic diarrhea

**DOI:** 10.1371/journal.pone.0204691

**Published:** 2018-09-27

**Authors:** Anna-Lena Ziese, Jan S. Suchodolski, Katrin Hartmann, Kathrin Busch, Alexandra Anderson, Fatima Sarwar, Natalie Sindern, Stefan Unterer

**Affiliations:** 1 Clinic of Small Animal Medicine, Centre for Clinical Veterinary Medicine, LMU Munich, Munich, Germany; 2 Gastrointestinal Laboratory, Department of Small Animal Clinical Sciences, Texas A&M University, TAMU, College Station, Texas, United States of America; Universidade Federal do Rio de Janeiro, BRAZIL

## Abstract

**Introduction:**

The impact of probiotics on dogs with acute hemorrhagic diarrhea syndrome (AHDS) has not been evaluated so far. The study aim was to assess the effect of probiotic treatment on the clinical course, intestinal microbiome, and toxigenic *Clostridium perfringens* in dogs with AHDS in a prospective, placebo-controlled, blinded trial.

**Methods:**

Twenty-five dogs with AHDS with no signs of sepsis were randomly divided into a probiotic (PRO; Visbiome, ExeGi Pharma) and placebo group (PLAC). Treatment was administered for 21 days without antibiotics. Clinical signs were evaluated daily from day 0 to day 8. Key bacterial taxa, *C*. *perfringens* encoding *NetF* toxin and enterotoxin were assessed on days 0, 7, 21.

**Results:**

Both groups showed a rapid clinical improvement. In PRO a significant clinical recovery was observed on day 3 (p = 0.008), while in PLAC it was observed on day 4 (p = 0.002) compared to day 0. Abundance of *Blautia* (p<0.001) and *Faecalibacterium* (p = 0.035) was significantly higher in PRO on day 7 compared to day 0, while in PLAC the abundance of *Faecalibacterium* was not significantly higher on any study day and *Blautia* (p = 0.016) was only significantly higher on day 21 compared to day 0. Abundance of *C*. *perfringens* was significantly lower on day 7 (p = 0.011) compared to day 0 in PRO but not in PLAC. Enterotoxin genes were significantly lower in PRO on day 21 (p = 0.028) compared to PLAC. Fecal samples of 57% of all dogs were positive for *netF* toxin genes on day 0 and the abundance was significantly lower on day 7 compared to day 0 in PRO (p = 0.016) and PLAC (p = 0.031).

**Conclusion:**

The probiotic treatment was associated with an accelerated normalization of the intestinal microbiome. Dogs with aseptic AHDS showed a rapid decrease of *netF* toxin genes and fast clinical recovery in both groups under symptomatic treatment without antibiotics.

## Introduction

Acute hemorrhagic diarrhea syndrome (AHDS) is a common complaint in dogs presented to primary care veterinarians. The etiology is not fully understood, but there is strong evidence that *C*. *perfringens* and its toxins play a role in the pathogenesis and are responsible for the intestinal lesions in most dogs diagnosed with AHDS [1]. An increase in fecal abundance of enterotoxigenic *C*. *perfringens* has been associated with acute non-hemorrhagic as well as with hemorrhagic diarrhea [2, 3]. Nevertheless, there was no difference found in severity of clinical or laboratory parameters between dogs with AHDS that were either positive or negative for *C*. *perfringens* encoding enterotoxin [3]. Recently, novel pore-forming toxins designated as *NetE* and *NetF* were identified in a *C*. *perfringens* type A strain isolated from a dog with acute hemorrhagic diarrhea, and the cytotoxic effect of *NetF* could be demonstrated *in vitro* [4]. In addition, there is a significantly higher prevalence of *C*. *perfringens* encoding *NetF* toxin (*netF*) in canine AHDS isolates compared to undifferentiated canine diarrheal isolates [4], and a preliminary study also showed a significant higher abundance of *netF* in dogs with AHDS compared to healthy dogs or dogs with parvovirosis [5].

The clinical picture of AHDS is characterized by acute onset of hemorrhagic diarrhea, lethargy, dehydration, and anorexia. Due to massive fluid loss, dogs with AHDS quickly develop hypovolemia, which can be potentially life threatening when untreated. Usually, a rapid clinical improvement under symptomatic treatment with aggressive fluid therapy, antiemetic therapy, analgetics, and gastrointestinal diet can be seen. Short-term prognosis is considered good after successful treatment of hypovolemia, while long-term consequences of the severe mucosal damage in dogs with AHDS are currently not known. Two individual studies have shown that treatment with antibiotics has no significant influence on mortality rate, duration of hospitalization, and clinical signs, and antibiotic treatment should be restricted to dogs with signs of systemic inflammatory response syndrome (SIRS) or inadequate response to symptomatic therapy [6, 7]. This is even more of interest since antibiotic treatment can cause acute alterations in the intestinal microbiome and some bacterial taxa even remain altered for months after antibiotic treatment [8, 9]. Moreover, inappropriate use of antibiotics promotes the development of antimicrobial resistance, which poses a major problem in health care [10].

Dogs with AHDS have alterations in the intestinal microbiome, for example increases in *C*. *perfringens*-like sequences and *Fusobacteria* and decreases in *Actinobacteria* and members within the *Firmicutes* (*Ruminococcaceae*, *Blautia* spp.) [11, 12].

Recently, a quantitative PCR-based dysbiosis index (DI) was developed to identify dysbiosis in canine fecal samples and this assay also allows to track microbiota changes over time [13]. The DI quantifies the abundance of total bacteria as well as of seven bacterial taxa (i.e. *Faecalibacterium*, *Turicibacter*, *E*. *coli*, *Streptococcus*, *Blautia*, *Fusobacterium* and *C*. *hiranonis*) shown to be altered in dogs with gastrointestinal disease in previous sequencing and qPCR-based studies. A recent study in dogs with multicentric lymphoma showed that the DI reflects the microbiota dysbiosis similarly as whole microbiota analysis by 16S rRNA gene sequencing [14]. The DI has been shown to be reproducible and provides a reference interval, based on assessment of the fecal microbiota of 95 healthy dogs [13]. The DI is expressed as a single numeric value and has shown to be negative in healthy dogs (mean DI -4.8) and positive in dogs with gastrointestinal disease (mean DI 3.3 in dogs with chronic enteropathy) indicating dysbiosis. However, it has been shown that there is an overlap of dysbiosis patterns between healthy dogs and dogs with gastrointestinal disease, which is why dogs with gastrointestinal disease may have normal microbiota and therefore negative DI.

Currently it is unclear whether the microbiota changes are in part causal or an effect of the disease, but it is believed that intestinal dysbiosis plays a role in the pathophysiology of acute and chronic disorders. Thus, a rapid normalization of microbiota dysbiosis might be beneficial.

Probiotics are orally administered live microorganisms which, when administered in adequate amounts, confer a health benefit on the host (FAO/WHO 2001). The exact mechanisms of probiotics are presently not fully understood, but several studies suggest that probiotics have different beneficial effects on host health, such as immunomodulation, anti-inflammatory properties or competition for nutrients or adhesion sites with potential pathogens [15, 16]. The effects of a probiotic possibly depend on the probiotic strain, mixture and concentration. A previous study for instance showed varying microbial changes in kenneled dogs depending on the dosage of the probiotic treatment [17]. Several studies showed an improvement in clinical signs by probiotic treatment such as causing significant decreased incidence of diarrhea in sheltered dogs and a significant reduction in duration of uncomplicated acute diarrhea in dogs [18–20]. Dogs with idiopathic inflammatory bowel disease treated with probiotics in addition to standard therapy showed an enhancement of regulatory T-cell markers, normalization of dysbiosis, and up-regulated expression of tight junction proteins [21, 22]. One study investigated the effect of probiotic treatment on dogs with hemorrhagic diarrhea due to parvovirosis and observed a more rapid improvement in clinical signs as well as in leukocyte counts in dogs additionally treated with probiotics [23]. However, to our knowledge there are no studies on probiotic treatment in dogs with AHDS and its impact on clinical signs and intestinal microbiota.

Thus, the aim of this prospective, placebo-controlled, randomized, blinded study was to evaluate whether treatment with a probiotic has an impact on the clinical course, the intestinal microbiome, and the presence and abundance of *C*. *perfringens* and *C*. *perfringens* encoding enterotoxin and *NetF* toxin in dogs with AHDS that show no signs of sepsis.

## Material & methods

### Patients

**Fig 1:**
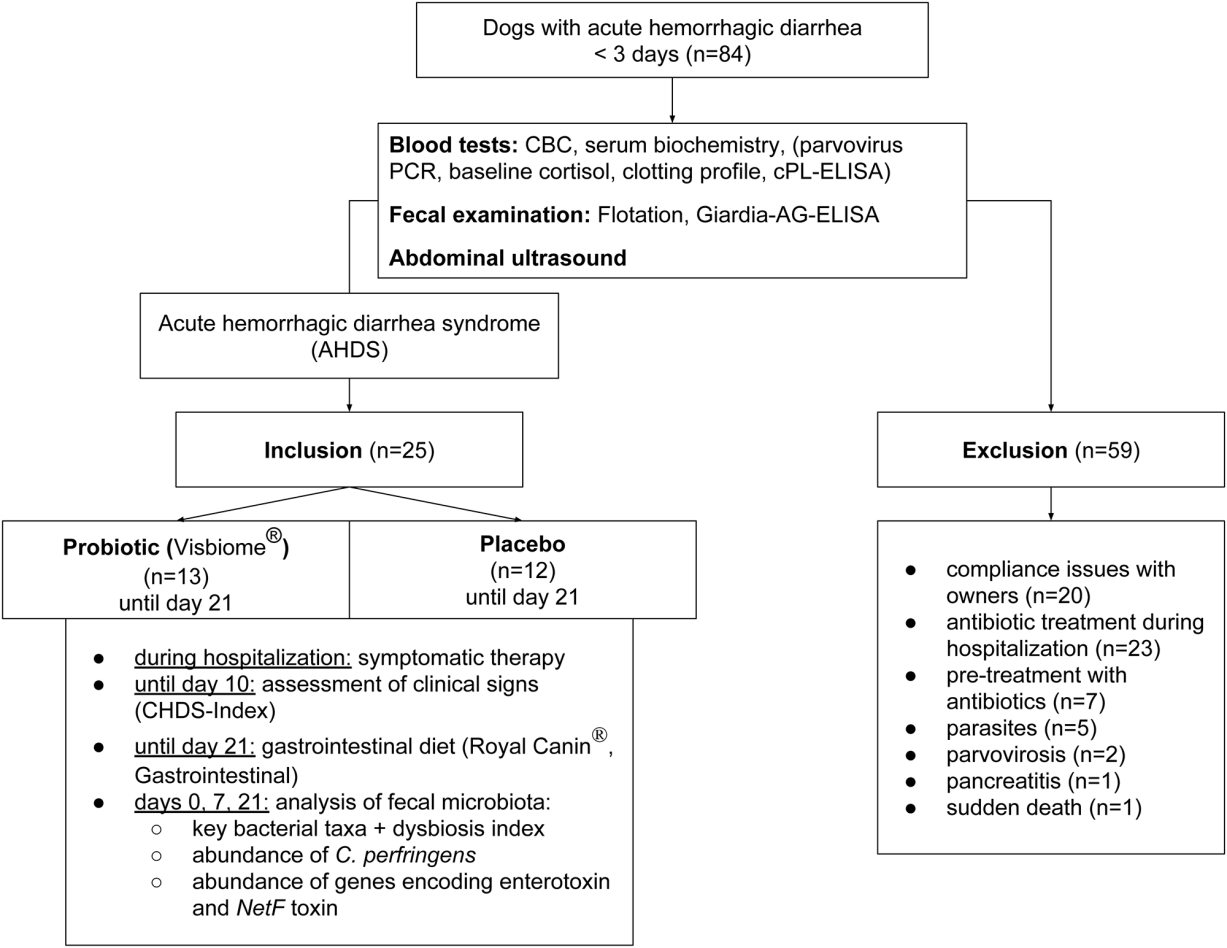
Standardized examination process and study protocol.

This study was a prospective, placebo-controlled, randomized, blinded treatment trial. It was conducted according to the German animal welfare law (approved by Ethics Commission, Centre for Clinical Veterinary Medicine, LMU Munich, Germany; reference number 9-20-06-13). Owners were informed about the purpose of the study, and all owners signed a written consent form. Between October 2013 and March 2015, 84 dogs were presented with acute hemorrhagic diarrhea to the Clinic of Small Animal Medicine, LMU University of Munich, Germany. AHDS was diagnosed by ruling out any disease that can potentially cause hemorrhagic diarrhea. Inclusion criterion to enter the study was the presence of acute onset of hemorrhagic diarrhea with or without vomiting lasting less than three days. Fig 1 illustrates the standardized examination process and exclusion criteria as well as the further course of the study as a flow-chart. Exclusion criteria were underlying diseases causing hemorrhagic diarrhea, pre-treatment with drugs known to cause mucosal irritation (e.g., non-steroidal anti-inflammatory drugs, corticosteroids, doxycycline) one week before presentation, or pre-treatment with antibiotics. Dogs diagnosed with AHDS but potential signs of sepsis (rectal temperature >39.50°C, white blood cell (WBC) count <4 or >25 x 10^9^/L, band neutrophil count >1.5 x 10^9^/L) were also excluded.

Therefore, a standardized history and physical examination were taken, and various tests performed.

#### Blood tests

A complete blood count (CBC), serum biochemistry profile, serum concentrations of pancreatic lipase immunoreactivity (Spec cPL, IDEXX Laboratories, Ludwigsburg, Germany) and clotting profiles if required (packed cell volume <40% or suspicious history or physical examination) were evaluated. A parvovirus PCR (IDEXX Laboratories, Ludwigsburg, Germany) was performed in dogs in which an infection was considered likely (young age, incomplete vaccination history, neutropenia). Baseline cortisol (IDEXX Laboratories, Ludwigsburg, Germany) concentration was measured to rule out Addison’s disease in dogs with lack of a stress leukogram and hyponatremia and/or hyperkalaemia.

#### Fecal examination

Flotation for nematodes and protozoan parasites (29.5% sodium nitrate flotation solution, Janssen-Cilag, Neuss, Germany) and a Giardia antigen ELISA (ProSpecT Giardia Microplate Assay; Remel Inc, Lenexa, KS) were evaluated.

#### Additional tests

Abdominal ultrasound was performed and urine specific gravity was measured in case of azotaemia.

### Treatment

Dogs (n = 25) that fulfilled the enrolment criteria were randomized by means of a computer-generated schedule into a probiotic group (PRO; n = 13) and a placebo group (PLAC; n = 12). Patients in PLAC received an orally administered placebo powder (maltose with trace amounts of silicon dioxide) packed in sachets and patients in PRO received a high potency, multi strain, orally administered probiotic powder every 24 hours for 21 days packed in sachets containing 450 billion cfu each. The placebo or probiotic powder was given orally either over food in dogs with appetite or diluted in water administered with a 5 mL syringe in anorectic patients. The probiotic mixture contained the following live bacterial strains: *Lactobacillus plantarum* DSM 24730, *Streptococcus thermophilus* DSM 24731, *Bifidobacterium breve* DSM 24732, *Lactobacillus paracasei* DSM 24733, *Lactobacillus delbrueckii* subsp. *bulgaricus* DSM 24734, *Lactobacillus acidophilus* DSM 24735, *Bifidobacterium longum* 120 DSM 24736, and *Bifidobacterium infantis* DSM 24737 in the specific combination which is currently sold under the brand Vivomixx in Continental Europe and Visbiome in the USA and Canada. According to the manufacturer, each strain in the probiotic blend was individually cultured and then filtered to separate the bacteria from the culture medium. The concentrated culture was then lyophilized for each strain separately. The finished product was shipped to the clinic and stored under refrigerated conditions to ensure product potency (4–8°C). Dosing was based on body weight: 225 billion colony forming units (cfu) for 1–10 kg dogs; 450 billion cfu for 10–20 kg dogs; 900 billion cfu for 20–40 kg dogs for 21 days on a daily basis.

Additional therapy during hospitalization was standardized and equal for both groups. It consisted of fluid therapy (crystalloids; amount depended on dehydration, maintenance demands, and ongoing losses) and antiemetics (maropitant 1 mg/kg SC q24 h; Cerenia, Pfizer Pharma GmbH) in case of vomiting. Analgetics were administered if required (buprenorphine 0.01 mg/kg intravenous every 6 hours or every 8 hours; Vetergesic Multidose, Patheon UK, Swindon, UK). A gastrointestinal diet (Royal Canin, Gastro Intestinal) was fed during hospitalization and owners were instructed to continue the diet until day 21 at home.

### Evaluation of clinical signs

Day 0 was defined as the day of clinical presentation and study inclusion. For eight days, clinical signs were assessed and quantified by a clinician during hospitalization or the owner at home using the canine hemorrhagic diarrhea severity index (CHDSI, Table 1). The CHDSI includes the parameters activity, appetite, vomiting (times/day), fecal consistency, defecation (times/day), and admixture of blood in the stool. Each parameter was scored from 0 to 3, and the sum of scores yielded a total cumulative score. On days with no bowel movement, the fecal consistency and defecation (times/day) were scored as zero. Clinicians as well as owners were blinded to the treatment. Only dogs showing normal activity and appetite, no vomiting, no dehydration, and no watery diarrhea (only dogs with normal, slightly soft or very soft fecal consistency; equivalent to a score of 0–2 according to the CHDSI) were discharged from the hospital.

**Table 1:** Criteria for assessment of the CHDS index (canine hemorrhagic diarrhea severity-index).

Parameter	0	1	2	3
Activity	Normal	Mildly reduced	Moderately reduced	Severely reduced
Appetite	Normal	Mildly reduced	Moderately reduced	Severely reduced
Vomiting	0	1x/day	2-3x/day	>3/day
Fecal consistency	Normal	Slightly soft	Very soft	Watery
Defecation	1x/day	2-3x/day	4-5x/day	>5/day
Blood admixtures	No	Mild	Moderate	Predominantly

Total score: 0–3: clinically insignificant; 4–5: mild; 6–8: moderate; >9: severe AHDS

### Analysis of fecal microbiota

Fecal samples collected on day 0, day 7, and day 21 were used for analysis of the fecal microbiota. Fecal DNA was extracted as described previously [13]. Briefly, an aliquot of 100 mg (wet weight) of each fecal sample was extracted by a bead-beating method using a MoBio Power soil DNA isolation kit (MoBio Laboratories, USA) following the manufacturer’s instructions. Quantitative PCR assays (qPCR) for key bacterial taxa that are altered in dogs with gastrointestinal disease (i.e., total bacteria, *Faecalibacterium*, *Turicibacter*, *Escherichia coli*, *Streptococcus*, *Blautia*, *Fusobacterium* and *Clostridium hiranonis*) were performed as previously described [13, 24]. PCR was also used to quantify the abundance of *C*. *perfringens* and the abundance of the genes encoding enterotoxin as described previously [2]. For quantification of genes encoding *netF*, primers were used as described previously [4]. The oligonucleotide sequences of primers and probes, and respective annealing temperatures are summarized in S1 Table. PCR conditions were 95°C for 20 seconds, 40 cycles at 95°C for 5 seconds, and 10 seconds at the optimized annealing temperature. For probe based assays, the mastermix consisted of 10 μL of TaqMan reaction mixtures containing 5 μL of TaqMan Fast Universal PCR master mix (2×), No AmpErase UNG (Applied Biosystems), 1 μL of water, 0.4 μL of each primer (final concentration: 400 nM), 0.2 μL of the probe (final concentration: 200 nM), 1 μL of 1% bovine serum albumin (BSA, final concentration: 0.1%), and 2 μL of DNA (1: 10 or 1: 100 dilution). For SYBR based assays PCR conditions were 95°C for 2 minutes, and 40 cycles at 95°C 5 seconds and 10 seconds at the optimized annealing temperature (S1 Table) with 10 μL of SYBR-based reaction mixtures containing 5 μL of SsoFast EvaGreen supermix (Biorad Laboratories), 1.6 μL of water, 0.4 μL of each primer (final concentration: 400 nM), 1 μL of 1% BSA (final concentration: 0.1%), and 2 μL of DNA (1:10 or 1:100 dilution). The qPCR results were expressed as the log amount of DNA (fg) for each bacterial group/10 ng of isolated total DNA. The results of the qPCR assays were statistically analyzed for individual taxa as well as expressed as single numerical value, the Dysbiosis Index (DI). A negative DI indicates normobiosis, whereas a positive DI indicates dysbiosis [13].

### Statistical analysis

The data for the qPCR assays, the CHDSI and signalment (age, gender, weight, breed) were tested for normal distribution using D’Agostino & Pearson omnibus normality tests. Group comparisons of data for the qPCR assays were performed using either an unpaired t-test or Mann-Whitney tests as appropriate. Comparisons within a group between time points were performed using either a paired t-test or Wilcoxon matched-pairs signed rank test as appropriate. Friedman test and Dunn’s multiple comparison test were used for comparison of CHDSI between time points among groups. Chi-square test was used for comparing proportions of dogs positive for *netF* toxin gene and enterotoxin gene. Significance was set at p<0.05. All statistics were performed in GraphPad Prism 7.0c (GraphPad Software Inc., San Diego, USA).

## Results

### Animals

Of the 84 dogs presented with hemorrhagic diarrhea during the study period, 59 patients were excluded from the study, due to various reasons (Fig 1). Seven of 84 dogs (8.3%) were excluded because they were pre-treated with antibiotics at time of presentation. Twenty-three (27.4%) were excluded because they were treated with antibiotics during hospitalization due to either signs of sepsis (n = 15/84; 17.9%), cystitis (n = 2/84; 2.4%), colitis (1/84; 1.2%), or unknown reasons (n = 5/84; 6.0%). Twenty dogs (23.8%) needed to be excluded because of compliance issues with owners (i.e., dogs would have fulfilled the inclusion criteria but owners were not willing to participate in the study initially or did not comply fecal sample collection during the study). Five dogs (6.0%) were tested positive for various parasites (e.g. *Toxacara canis*, *Giardia*, *Cryposporidia*), two dogs (2.4%) had a positive PCR result for parvovirosis, one dog (1.2%) was diagnosed with pancreatitis, and in one dog (1.2%) sudden death occurred during initial examination.

Twenty-five of 84 dogs (29.8%) were diagnosed with acute hemorrhagic diarrhea syndrome and eligible to enter the study. They were randomly divided into two groups, PRO (n = 13) and PLAC (n = 12) by means of a computer-generated schedule. The mean (SD) age in PRO was 6.0 years (3.9) while it was 5.5 years (3.9) in PLAC. No significant differences in age (p = 0.748), gender (p = 0.561), body weight (p = 0.397) or breed distribution (p = 0.390) were found between the two groups.

### CHDS index (CHDSI)

**Fig 2:**
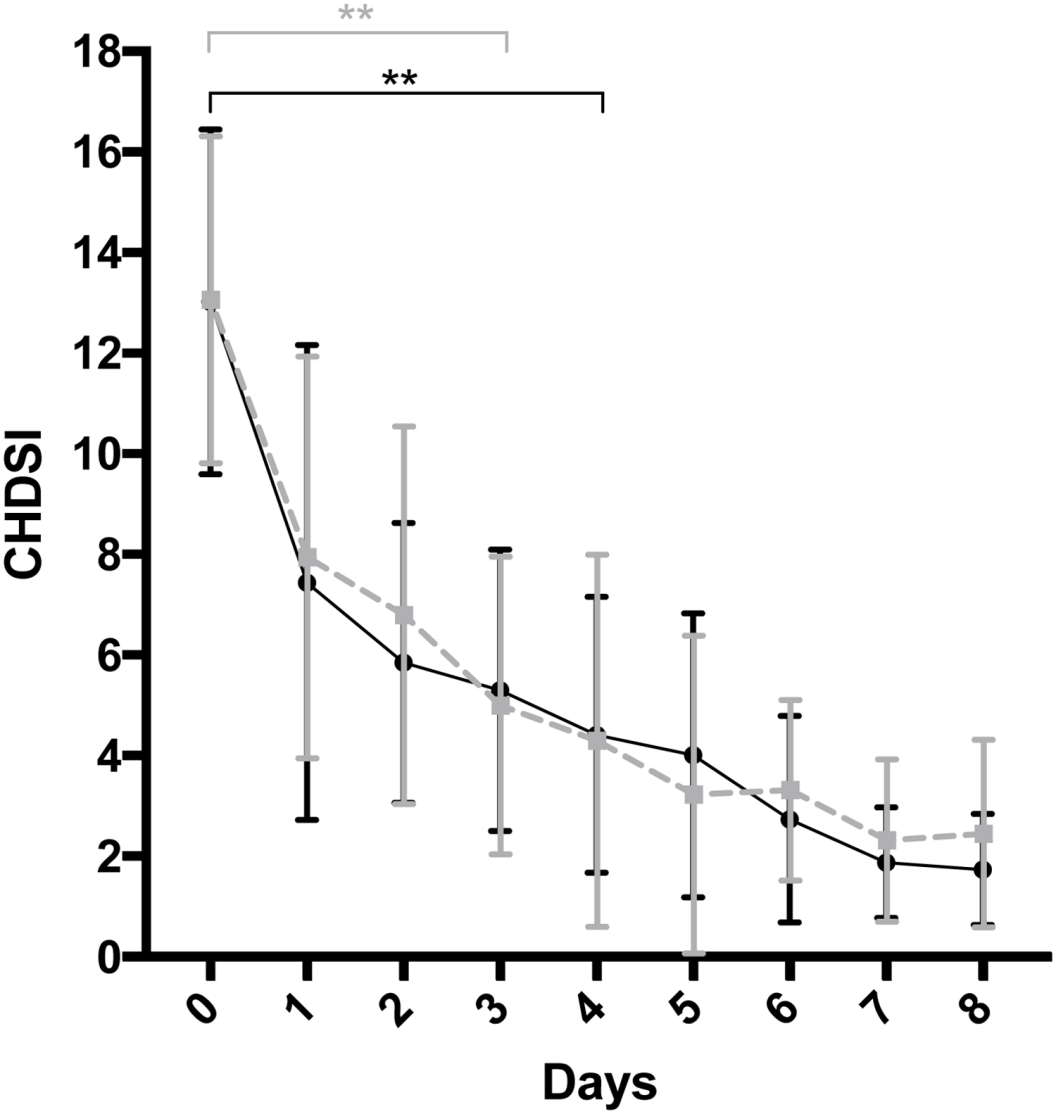
Assessment of clinical signs with the canine hemorrhagic diarrhea severity index (CHDSI). Index includes the parameters activity, appetite, vomiting (times/day), stool consistency, defecation (times/day) and admixture of blood in the stool. Each parameter is scored from 0 to 3, and the sum of scores yielded a total cumulative score. PRO = probiotic group (grey, dashed); PLAC = placebo group (black). Error bars show mean + standard deviation. In PRO the first day with a significantly lower CHDSI compared to day 0 was day 3 (p = 0.008) while in PLAC the first day with a significantly lower CHDSI compared to day 0 was day 4 (p = 0.002).

On day 0, mean (SD) CHDSI in PRO was 13 (3.2) and in PLAC it was 13 (3.4), which was not significantly different between groups (p = 0.980) (Fig 2). Compared to day 0, there was a significantly lower CHDSI observed on day 3 (p = 0.008) in PRO with mean CHDSI of 5.0 (3.0). In PLAC there was a significant lower CHDSI observed on day 4 (p = 0.002) compared to day 0 with mean CHDSI of 5.2 (2.8). After day 6, the mean CHDSI stayed below 3 in both groups, indicating clinically insignificant signs according to the CHDSI. There were no further significant differences observed between PRO and PLAC on any study day.

### Fecal microbiota

**Fig 3:**
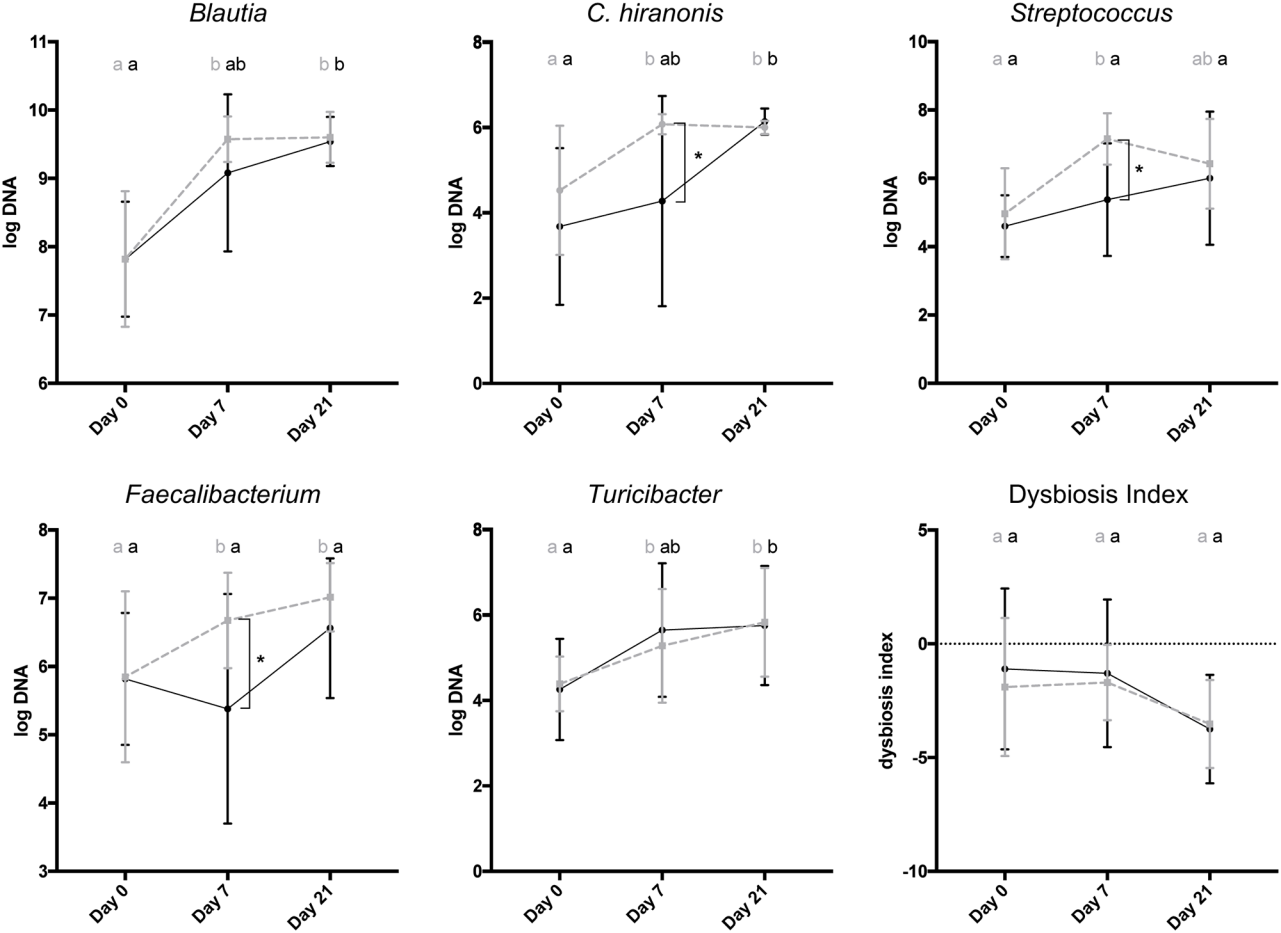
Abundance of *Blautia*, *C*. *hiranonis*, *Streptococcus*, *Faecalibacterium*, *Turicibacter* and dysbiosis index. PRO = probiotic group (grey, dashed); PLAC = placebo group (black). A negative DI indicates normobiosis, whereas a positive DI indicates dysbiosis. Error bars show mean + standard deviation. Days not sharing a common superscript are significantly different from each other (p = <0.05). Asterisk indicates significant difference (p = <0.05) between groups.

On day 0, the abundances of *Faecalibacterium*, *Turicibacter*, *E*. *coli*, *Streptococcus*, *Blautia*, *Fusobacterium*, and *C*. *hiranonis* were not significantly different between PRO and PLAC (Table 2; Fig 3). In PRO the abundances of *Blautia* (p<0.001), *C*. *hiranonis* (p = 0.001), *Streptococcus* (p = 0.001), *Faecalibacterium* (p = 0.035) and *Turicibacter* (p = 0.037) were significantly higher on day 7 compared to day 0. In contrast, in PLAC there was a significantly higher abundance of *Blautia* (p = 0.016), *C*. *hiranonis* (p = 0.014) and *Turicibacter* (p = 0.008) only seen on day 21 compared to day 0. There was no significantly higher abundance of *Streptococcus* and *Faecalibacterium* in PLAC on any study day compared to day 0. Additionally, dogs in PRO had a significantly higher abundance of *C*. *hiranonis* (p = 0.014), *Streptococcus* (p = 0.013) and *Faecalibacterium* (p = 0.018) compared to PLAC on day 7. No significant changes over time within groups or between groups were observed for *E*. *coli* and *Fusobacterium* (Table 2). The baseline dysbiosis index (DI) was not significantly different (p = 0.567) between PRO and PLAC (Fig 3). On day 0, 4/13 dogs (31%) in PRO and 4/10 dogs (40%) in PLAC had a DI above 0. The DI showed a decreasing tendency with 3/13 dogs (23%) in PRO, 3/12 dogs (25%) in PLAC having a DI above 0 on day 7. After day 7, only one dog in PLAC had a DI above 0 on day 21, and there was no significant difference between PRO and PLAC or between time points among groups.

**Table 2:** Abundance of bacterial groups in dogs with acute hemorrhagic diarrhea syndrome.

	PRO			PLAC		
	Day 0	Day 7	Day 21	Day 0	Day 7	Day 21
*E*. *coli*	5.91 (1.70) ^a^	5.30 (1.54) ^a^	5.49 (1.18) ^a^	6.60 (1.46) ^a^	5.80 (1.86) ^a^	5.43 (1.70) ^a^
*Faecalibacterium*	5.85 (1.25) ^a^	6.68 (0.70) ^b^	7.02 (0.50) ^b^	5.82 (0.97) ^a^	5.38 (1.68) ^a^	6.56 (1.02) ^a^
*Turicibacter*	4.39 (0.64) ^a^	5.28 (1.33) ^b^	5.84 (1.27) ^b^	4.26 (1.18) ^a^	5.64 (1.56) ^ab^	5.75 (1.39) ^b^
*Streptococcus*	4.96 (1.33) ^a^	7.15 (0.75) ^b^	6.42 (1.31) ^ab^	4.61 (0.90) ^a^	5.39 (1.65) ^a^	6.01 (1.95) ^a^
*Blautia*	7.82 (0.99) ^a^	9.57 (0.33) ^b^	9.60 (0.37) ^b^	7.82 (0.84) ^a^	8.35 (1.15) ^ab^	9.24 (0.36) ^b^
*Fusobacterium*	9.08 (1.08) ^a^	8.81 (0.84) ^a^	9.00 (0.62) ^a^	8.73 (1.09) ^a^	9.41 (1.04) ^a^	9.34 (0.78) ^a^
*C*. *hiranonis*	4.53 (1.52) ^a^	6.08 (0.24) ^b^	6.01 (0.16) ^b^	3.68 (1.83) ^a^	4.27 (2.46) ^ab^	6.13 (0.30) ^b^
*C*. *perfringens*	6.98 (1.17) ^a^	5.80 (1.15) ^b^	4.79 (1.41) ^b^	6.52 (1.37) ^a^	5.17 (2.40) ^a^	5.97 (1.45) ^a^

PRO = probiotic group; PLAC = placebo group; values represent mean (SD) log DNA/g feces.

Columns within groups not sharing a common superscript are significantly different from each other (p<0.05).

### C. perfringens

**Fig 4:**
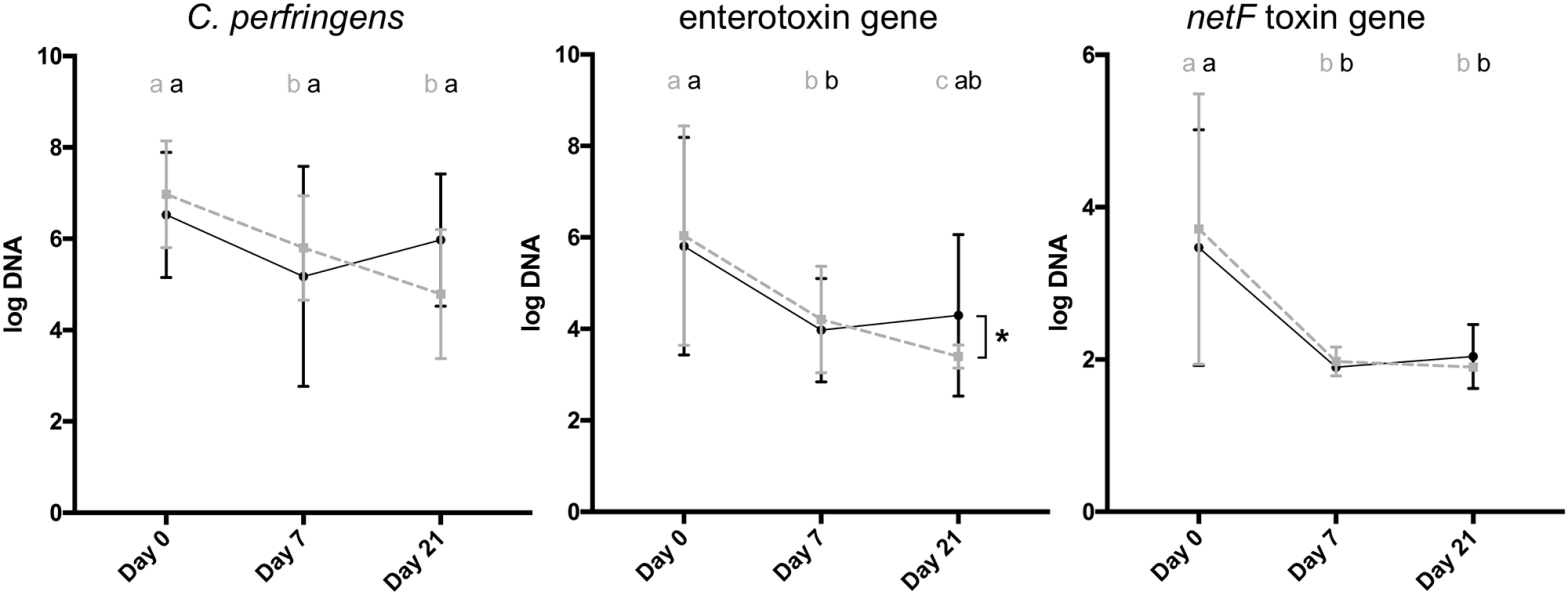
Abundance of *C*. *perfringens*, *C*. *perfringens* encoding enterotoxin and *NetF* toxin. PRO = probiotic group (grey, dashed); PLAC = placebo group (black). Error bars indicate mean + standard deviation. Days not sharing a common superscript are significantly different from each other (p<0.05). Asterisk indicates significant difference (p<0.05) between groups.

All dogs were positive for *C*. *perfringens* on all days. On day 0, the abundance of *C*. *perfringens* was not significantly different (p = 0.376) between PRO and PLAC (Fig 4). The abundance of *C*. *perfringens* was significantly lower on day 7 (p = 0.011) compared to baseline in PRO. In contrast, in PLAC *C*. *perfringens* were not significantly lower on any study day compared to baseline.

### *C*. *perfringens* strains encoding enterotoxin

On day 0, the abundance (p = 0.842) as well as the number of dogs positive for enterotoxin genes (10/13 positive in PRO vs. 7/10 in PLAC; p = 0.708) were not significantly different between PRO and PLAC (Fig 4). On day 7, the abundance of enterotoxin genes was significantly lower compared to baseline in both PRO (p = 0.016) and PLAC (p = 0.016) and also number of dogs positive for enterotoxin genes was not significantly different between groups (7/13 positive in PRO vs. 6/12 in PLAC; p = 0.695). On day 21, the abundance of enterotoxin genes was significantly lower in PRO compared to PLAC (p = 0.028). Proportions of dogs positive for enterotoxin encoding *C*. *perfringens* strains were also significantly lower in dogs receiving probiotic on day 21 (1/10 positive in PRO vs. 5/8 in PLAC; p = 0.019).

### *C*. *perfringens* strains encoding *NetF* toxin (*netF*)

On day 0, a total of 13/23 (57%) dogs were positive for *netF* and there was no significant difference in number of dogs positive for *netF* between groups (7/13 positive in PRO vs. 6/10 positive in PLAC, p = 0.768). Additionally, the baseline abundance of *netF* was not significantly different (p = 0.764) between PRO and PLAC (Fig 4). The abundance of *netF* was significantly lower on day 7 compared to baseline in both PRO (p = 0.016) and PLAC (p = 0.031). After day 7 all dogs except one in PLAC on day 21, were below the detection limit of the PCR assay for *netF*. There was no significant difference in abundance or number of dogs positive for *netF* between groups on any study day.

## Discussion

The aim of this study was to evaluate whether probiotic treatment has an impact on the clinical course, the intestinal microbiome and the abundance of *C*. *perfringens* and toxigenic *C*. *perfringens* in dogs with AHDS. Therefore, dogs with hemorrhagic diarrhea lasting less than three days were included in this trial. Dogs with an underlying disease possibly responsible for hemorrhagic diarrhea or dogs with potential signs of sepsis at clinical presentation or during the study were excluded. Dogs in the probiotic group received a high potency, multi strain, orally administered probiotic powder, which was chosen based on previous studies demonstrating upregulated expression of tight junction proteins and clinical response in dogs with IBD [21, 22].

Acute hemorrhagic diarrhea syndrome is characterized by its self-limiting rapid course and has a good short-term prognosis when treated symptomatically [7, 25]. We observed that all dogs that fulfilled the inclusion criteria indeed showed a rapid clinical improvement within a few days. Based on the CHDSI, dogs in both groups were considered to have mild signs already after 4 days and both groups had clinically insignificant signs after only 6 days (mean CHDSI below 3). These results correspond to the findings in previous studies [7, 25]. We observed that a significant recovery in PRO was already seen on day 3, while in PLAC it was seen on day 4 compared to baseline. No further differences in severity or duration of clinical signs between PRO and PLAC were seen. Since dogs with aseptic AHDS usually show a rapid clinical improvement, more significant differences in clinical signs between treatment groups might have been difficult to detect even if they were documented on a daily basis as performed in this study.

Many practitioners still administer antibiotics to dogs with acute hemorrhagic diarrhea even if patients do not show any signs of sepsis. In our study, 15 of the 84 dogs (17.9%) with acute hemorrhagic diarrhea were not included in the study since they showed signs that potentially reflect sepsis. A definitive diagnosis of sepsis in dogs with AHDS is difficult, since there is a significant overlap concerning criteria for sepsis and hypovolemia. Individual dogs with AHDS are still at risk to develop sepsis, likely due to bacterial translocation because of a damaged intestinal barrier. Of particular interest is the fact that the dogs in our study that had no signs of sepsis showed a rapid improvement under symptomatic therapy, and all dogs returned to a normal stool consistency and frequency within a few days without antibiotic treatment. This emphasizes the findings of previous studies, which showed that antibiotic therapy yields no advantages compared to symptomatic therapy in dogs with acute diarrhea as long as the patients do not show signs of sepsis [6, 7]. This is even more of interest, since bacterial resistances are an increasing problem in public health care and unnecessary use of antibiotics should be avoided [26, 27].

The intestinal tract of healthy dogs is inhabited by a diverse intestinal microbiota with *Firmicutes*, *Bacteroidetes*, *Proteobacteria*, and *Fusobacteria* being the predominant bacterial phyla. The intestinal microbiota plays an important role in host health by contributing to many different pathways, such as producing metabolites like short chain fatty acids or by participating in bile acid conversion [28–31]. Moreover, a crosstalk between gut microbiota and host immune cells exists, enabled by microbial-derived metabolites and bacterial surface molecules [32]. It has been shown that dogs with acute hemorrhagic diarrhea have alterations in their intestinal microbiome. Bacterial taxa most commonly altered include increases in *C*. *perfringens* and *E*. *coli*, and decreases in *Blautia*, *Turicibacter*, *Faecalibacterium*, and *Streptococcus* spp. compared to healthy dogs [11, 12, 33]. Recently, a quantitative PCR-based dysbiosis index (DI) was developed to quantify these specific bacterial groups and combine them numerically into one single number to assess fecal dysbiosis in canine fecal samples [13]. A negative DI indicates normobiosis, whereas a positive DI indicates dysbiosis. On day 0, 31% of dogs in PRO and 40% of dogs in PLAC had a DI above zero, indicating intestinal dysbiosis. Overall, the DI showed a similar tendency to decrease in both groups reflecting normalization of the intestinal microbiota. Within 21 days, all dogs except one dog in PLAC returned to a negative DI indicating intestinal normobiosis. These findings suggest that AHDS is a self-limiting syndrome not only regarding clinical signs but also regarding the intestinal microbiome.

Species belonging to the genus *Blautia* are involved in glucose metabolism, producing metabolites like acetate, ethanol, hydrogen, lactate and succinate [34], and *Blautia* represent about 8.9–25.2% (median 14.0%) of the intestinal bacteria [12]. Studies in humans have shown that a decreased abundance of *Blautia* is accompanied with negative effects on host health, such as negative prognostic factors in early-stage breast cancer or higher graft-versus-host disease related mortality [35, 36]. *C*. *hiranonis* is considered to be a beneficial bacterium that plays a role in bile acid conversion [28] while *Turicibacter* is likely to play a role in the metabolism of butyric acid [37]. *Faecalibacterium* was shown to have anti-inflammatory properties and human inflammatory bowel disease and infectious colitis were associated with low counts of *Faecalibacterium* [38, 39]. *Blautia* have a mean abundance of 9.7 log DNA, *C*. *hiranonis* a mean abundance of 6.4 log DNA, *Faecalibacterium* a mean abundance of 6.2 log DNA and *Turicibacter* a mean abundance of 6.1 log DNA per gram of feces as described in 95 healthy dogs using the same PCR techniques [13]. In veterinary medicine, previous studies observed a significant decrease in *Blautia*, *Faecalibacterium* and *Turicibacter* in dogs with acute hemorrhagic and non-hemorrhagic diarrhea [3, 11, 12]. Consistent to those previous findings, in our study we observed a reduced mean abundance of *Blautia*, *Faecalibacterium* and *Turicibacter* (Table 2) on day 0 according to the reference intervals set by AlShawaqfeh et al. In dogs treated with the probiotic we observed a higher abundance of *Blautia*, *Faecalibacterium*, *Turicibacter*, *C*. *hiranonis* and *Streptococcus* on day 7 compared to baseline while in dogs treated with placebo a higher abundance was only seen on day 21 or on no study day at all (Fig 3).

*C*. *perfringens* is a commensal of the intestinal tract and can be found in up to 76% in the faces of healthy non-diarrheic dogs [2, 40, 41]. It has been shown that dogs with acute hemorrhagic and non-hemorrhagic diarrhea have a significantly higher abundance of *C*. *perfringens* [2], while dogs with IBD do not have a significantly higher abundance compared to healthy dogs [11]. Moreover, the abundance of *C*. *perfringens* encoding enterotoxin and the prevalence of dogs positive for enterotoxin itself via ELISA toxin immunoassay are significantly higher in dogs with acute diarrhea than in healthy dogs [2]. However, *C*. *perfringens* enterotoxin is unlikely to be the primary cause of AHDS, since there was no difference found in severity of clinical signs, duration of hospitalization or laboratory parameters between dogs positive or negative for *C*. *perfringens* encoding enterotoxin based on PCR assays [3]. Additionally, there was no difference seen in dogs being positive or negative for enterotoxin itself based on ELISA toxin immunoassay data [3]. In this study, we detected a significantly lower abundance of *C*. *perfringens* in PRO compared to baseline on day 7, whereas there was no significant lower abundance in PLAC on any day compared to day 0. Proportions of dogs positive as well as the abundance of *C*. *perfringens* encoding enterotoxin were significantly lower in dogs receiving probiotic on day 21 in comparison to dogs that received placebo. These findings suggest that probiotic treatment in this study was associated with an increase of beneficial bacteria such as *Blautia*, *C*. *hiranonis*, *Streptococcus*, *Faecalibacterium* and *Turicibacter* and an accelerated decrease of possible pathogenic bacteria like *C*. *perfringens*. We did not examine the exact mechanisms of how the probiotic mixture affected the intestinal microbiota in this study. Probiotic bacterial strains can modulate the immune system and enhance intestinal barrier function through different mechanisms of action. Studies reported a response of the gut associated lymphoid tissue (GALT) following the administration of *L*. *plantarum* and *L*. *acidophilus* [42, 43]. Also antibody production such as IgA from plasma cells, which protect the host by binding several antigens were observed following treatment with *B*. *lactis* strains [44]. Moreover, previous studies observed that Lactobacillus strains (*L*. *acidophilus* and *L*. *fermentum*) are able to inhibit the growth of *C*. *perfringens* in vitro. This inhibition was not only caused by lowering the pH level, as *C*. *perfringens* is a pH sensitive bacterium, but also likely due to bacteriocin production by *L*. *acidophilus* and *L*. *fermentum* [45, 46]. The probiotic mixture used in our study may be associated with an increase of beneficial bacteria and decrease of possible pathogenic bacteria due to similar mechanisms. The formula of the probiotic used in our study contains a higher concentration of bacteria compared to several other probiotic products, which may have promoted this effect. Further research is warranted to elucidate the mechanisms of action of probiotics in dogs with AHDS.

The novel pore-forming cytotoxic toxin *NetF* was recently isolated from a dog with acute hemorrhagic diarrhea [4]. The study observed a highly significant association between the presence of *C*. *perfringens* encoding *NetF* toxin and canine hemorrhagic diarrhea compared to undifferentiated enteritis and 75% of the isolates from hemorrhagic diarrhea were positive for *netF* [4]. In our study, we observed that 57% of all dogs that fulfilled the inclusion criteria were positive for *netF* at time of clinical presentation. *NetF* decreased rapidly, and after day 7 all dogs, except one dog in PLAC on day 21, were below the detection limit of the PCR assay. Since both groups showed a rapid decrease, it is likely that the *NetF* toxemia in dogs with hemorrhagic diarrhea is self-limiting and parallels clinical improvement.

A limitation of this study is the small sample size of 25 dogs. Future studies with a higher sample size could provide more detailed data regarding the changes in microbiome and may also be able to detect more significant differences in the clinical course. Additionally, a more frequent analysis of fecal samples, especially in the first days after clinical presentation, could have offered a more precise insight on the course of the intestinal microbiome in dogs with AHDS treated with the probiotic. A possible limitation also may be that we did not perform a whole screening of the intestinal microbiome. Reasons for not performing a sequencing-based approach was that we employed reproducible qPCR assays that targeted those bacterial groups previously found to be altered in canine gastrointestinal disease, including AHDS. Additionally, we also did not perform a toxin immunoassay for *C*. *perfringens* enterotoxin, as the commercially available immunoassay has not been validated for use in dogs. To our knowledge, no immunoassay is available for measurement of *NetF* toxin.

In conclusion, the results of this study show that aseptic AHDS is characterized by its rapid self-limiting course regarding clinical signs and the presence of *netF* toxin genes. Both groups recovered quickly with a significant improvement on day 3 in the probiotic group and day 4 in placebo group compared to day of clinical presentation. Dogs receiving probiotic treatment also showed an accelerated normalization of *Blautia*, *C*. *hiranonis*, *Faecalibacterium*, and *Turicibacter* compared to dogs that were only treated symptomatically. Additionally, the abundance of *C*. *perfringens* encoding enterotoxin was significantly lower in dogs receiving probiotics.

## Supporting information

S1 Table Oligonucleotides primers/probes used in this study. (DOCX)
