# Correction: Effect of probiotic treatment on the clinical course, intestinal microbiome, and toxigenic *Clostridium perfringens* in dogs with acute hemorrhagic diarrhea

**DOI:** 10.1371/journal.pone.0280539

**Published:** 2023-01-12

**Authors:** Anna-Lena Ziese, Jan S. Suchodolski, Katrin Hartmann, Kathrin Busch, Alexandra Anderson, Fatima Sarwar, Natalie Sindern, Stefan Unterer

Following publication of this article [1], the authors provide revised text to clarify the composition of the probiotic mixture, sold under the brand Vivomixx in Continental Europe, which was used in the study. The correct fourth sentence of the Treatment subsection of the Materials and Methods is as follows:

According to the manufacturer, the probiotic mixture contained the following live bacterial strains: *Lactobacillus plantarum*, *Streptococcus thermophilus*, *Bifidobacterium breve*, *Lactobacillus paracasei*, *Lactobacillus delbrueckii* subsp. *bulgaricus*, *Lactobacillus acidophilus*, *Bifidobacterium longum*, and *Bifidobacterium infantis*; the authors’ research did not extend to confirm the bacterial composition information provided by the manufacturer.

The authors also provide the following additional information regarding the rationale for the time points used in the study: Day 0 served as a baseline to capture the microbial status before starting the treatment. Day 7 was chosen because previous clinic experience showed that most dogs with acute hemorrhagic diarrhea syndrome (AHDS) recover clinically within this time period. Day 21 was chosen since the probiotic or placebo was administered until this day.

The data underlying Figs 2–4 and Table 2 are missing from the list of Supporting Information. The authors provide the data as Supporting Information files with this notice. Where CHDSI was evaluated by more than one clinician, an average of the values was used for the statistical evaluation (Fig 2 of [1], S1 File).

With this correction, all relevant data are now provided.

## Supporting information

S1 File CHDSI data underlying Fig 2. (XLSX)

S2 File qPCR and Dysbiosis Index data underlying Fig 2, Fig 3, Table 2. (ZIP)
