# Supplementary material for: Effect of probiotic treatment on the clinical course, intestinal microbiome, and toxigenic Clostridium perfringens in dogs with acute hemorrhagic diarrhea
Source: PLoS One. 2018 Sep 27;13(9):e0204691. doi: 10.1371/journal.pone.0204691 (PMC6160196; doi:10.1371/journal.pone.0204691)
Supplement: S1 Table — (DOCX) [file pone.0204691.s001.docx]

| **S1 Table. Oligonucleotides primers/probes used in this study.** | |  |  |  |
| --- | --- | --- | --- | --- |
| qPCR primers/probe | Sequence (5’- 3’) | Target | Annealing (°C) | Reference |
|  |  |  |  |  |
| Forward | GAAGGCGGCCTACTGGGCAC | *Faecalibacterium* | 60 | [1] |
| Reverse | GTGCAGGCGAGTTGCAGCCT |  |  |  |
|  |  |  |  |  |
| Forward | KGGGCTCAACMCMGTATTGCGT | Fusobacteria | 51 | [2] |
| Reverse | TCGCGTTAGCTTGGGCGCTG |  |  |  |
|  |  |  |  |  |
| Forward | TCTGATGTGAAAGGCTGGGGCTTA | *Blautia* | 56 | [2] |
| Reverse | GGCTTAGCCACCCGACACCTA |  |  |  |
|  |  |  |  |  |
| Forward | CCTACGGGAGGCAGCAGT | Universal Bacteria | 59 | [3] |
| Reverse | ATTACCGCGGCTGCTGG |  |  |  |
|  |  |  |  |  |
| Forward | CAGACGGGGACAACGATTGGA | *Turicibacter* | 63 | [2] |
| Reverse | TACGCATCGTCGCCTTGGTA |  |  |  |
|  |  |  |  |  |
| Forward | GTTAATACCTTTGCTCATTGA | *E. coli* | 55 | [4] |
| Reverse | ACCAGGGTATCTAATCCTGTT |  |  |  |
|  |  |  |  |  |
| Forward | AGTAAGCTCCTGATACTGTCT | *C. hiranonis* | 50 | [5] |
| Reverse | AGGGAAAGAGGAGATTAGTCC |  |  |  |
|  |  |  |  |  |
| Forward | TTATTTGAAAGGGGCAATTGCT | *Streptococcus* | 54 | [6] |
| Reverse | GTGAACTTTCCACTCTCACAC |  |  |  |
|  | | |  |  |
| Forward | AACTATAGGAGAACAAAATACAATAG | *C. perfringens* enterotoxin gene | 55 | [7] |
| Reverse | TGCATAAACCTTATAATATACATATTC |  |  |  |
| Probe | FAM-TCTGTATCTACAACTGCTGGTCCA-TAMURA |  |  |  |
|  |  |  |  |  |
| Forward | CGCATAACGTTGAAAGATGG | *C. perfringens* 16S rRNA gene | 58 | [8] |
| Reverse | CCTTGGTAGGCCGTTACCC |  |  |  |
| Probe | FAM-TCATCATTCAACCAAAGGAGCAATCC-TAMURA |  |  |  |
|  |  |  |  |  |
| Forward | AACAATATGTACAGGTATAACT | *C. perfringens* *netF* toxin gene | 55 | [9] |
| Reverse | TTGATAGGTATAATATGGTTCT |  |  |  |

**References:**

1. Garcia-Mazcorro JF, Suchodolski JS, Jones KR, Clark-Price SC, Dowd SE, Minamoto Y, et al. Effect of the proton pump inhibitor omeprazole on the gastrointestinal bacterial microbiota of healthy dogs. FEMS Microbiol Ecol. 2012;80(3):624-36. doi: 10.1111/j.1574-6941.2012.01331.x. PMID: 22324305.

2. Suchodolski JS, Markel ME, Garcia-Mazcorro JF, Unterer S, Heilmann RM, Dowd SE, et al. The fecal microbiome in dogs with acute diarrhea and idiopathic inflammatory bowel disease. PLoS One. 2012;7(12):e51907. doi: 10.1371/journal.pone.0051907. PMID: 23300577.

3. Lubbs DC, Vester BM, Fastinger ND, Swanson KS. Dietary protein concentration affects intestinal microbiota of adult cats: a study using DGGE and qPCR to evaluate differences in microbial populations in the feline gastrointestinal tract. J Anim Physiol Anim Nutr (Berl). 2009;93(1):113-21. doi: 10.1111/j.1439-0396.2007.00788.x. PMID: 19386015.

4. Malinen E, Rinttila T, Kajander K, Matto J, Kassinen A, Krogius L, et al. Analysis of the fecal microbiota of irritable bowel syndrome patients and healthy controls with real-time PCR. Am J Gastroenterol. 2005;100(2):373-82. doi: 10.1111/j.1572-0241.2005.40312.x. PMID: 15667495.

5. Kitahara M, Sakamoto M, Benno Y. PCR detection method of *Clostridium scindens* and *C. hiranonis* in human fecal samples. Microbiol Immunol. 2001;45(3):263-6. PMID: 11345537.

6. Furet JP, Quenee P, Tailliez P. Molecular quantification of lactic acid bacteria in fermented milk products using real-time quantitative PCR. Int J Food Microbiol. 2004;97(2):197-207. doi: 10.1016/j.ijfoodmicro.2004.04.020. PMID: 15541806.

7. Gurjar AA, Hegde NV, Love BC, Jayarao BM. Real-time multiplex PCR assay for rapid detection and toxintyping of *Clostridium perfringens* toxin producing strains in feces of dairy cattle. Mol Cell Probes. 2008;22(2):90-5. doi: 10.1016/j.mcp.2007.08.001. PMID: 17890052.

8. Wise MG, Siragusa GR. Quantitative detection of *Clostridium perfringens* in the broiler fowl gastrointestinal tract by real-time PCR. Appl Environ Microbiol. 2005;71(7):3911-6. doi: 10.1128/AEM.71.7.3911-3916.2005. PMID: 16000804.

9. Gohari IM, Parreira VR, Nowell VJ, Nicholson VM, Oliphant K, Prescott JF. A novel pore-forming toxin in type A *Clostridium perfringens* is associated with both fatal canine hemorrhagic gastroenteritis and fatal foal necrotizing enterocolitis. PLoS One. 2015;10(4):e0122684. doi: 10.1371/journal.pone.0122684. PMID: 25853427.
